# Increased von Willebrand factor parameters in children with febrile seizures

**DOI:** 10.1371/journal.pone.0210004

**Published:** 2019-01-03

**Authors:** Astrid Pechmann, Sven Wellmann, Benjamin Stoecklin, Marcus Krüger, Barbara Zieger

**Affiliations:** 1 Department of Neuropediatrics and Muscle Disorders, Medical Center - University of Freiburg, Faculty of Medicine, University of Freiburg, Freiburg, Germany; 2 Department of Neonatology, University Children’s Hospital Basel UKBB, University of Basel, Basel, Switzerland; 3 Department of Neonatology, Municipal Hospital Munich Campus Harlaching and Schwabing, Germany; 4 Department of Pediatrics and Adolescent Medicine, Division of Pediatric Hematology and Oncology, Medical Center - University of Freiburg, Faculty of Medicine, University of Freiburg, Freiburg, Germany; Chinese Academy of Medical Sciences and Peking Union Medical College, CHINA

## Abstract

**Introduction:**

Primary blood coagulation and wound sealing are orchestrated by von Willebrand factor (VWF), a large multimeric glycoprotein. Upon release of arginine vasopressin (AVP), VWF containing high molecular weight multimers is secreted. By measuring copeptin, the C-terminal part of the AVP prohormone, we recently found strongly increased AVP levels in children with febrile seizures (FS) as compared to children with fever but without seizures. It is unknown if increased AVP levels in FS are of any biological function. Therefore, our a priori hypothesis was that children with FS have increased VWF parameters in parallel with higher AVP levels.

**Methods:**

We conducted a prospective, cross-sectional study of children aged between 6 months and 5 years. Children that presented at our emergency department with fever or a recent FS (within four hours) were evaluated to be included to the study. We measured serum copeptin and VWF parameters, including analyses of VWF:Antigen (WVF:Ag), VWF:collagen binding activity (VWF:CB) and VWF multimers in children with FS, febrile infections without seizures and additionally, in a non-febrile control group.

**Results:**

We included 54 children in our study, 30 with FS, 10 in the febrile control group, and 14 in the non-febrile control group. Serum copeptin levels were significantly higher in children with FS (median [IQR] 24.73 pmol/l [13.65–68.65]) compared to the febrile control group (5.66 pmol/l [4.15–8.07], p = 0.002) and the non-febrile control group (4.78 pmol/l [3.33–5.3], p<0.001). VWF:CB levels were also significantly higher in children with FS (VWF:CB 2.29 U/ml [1.88–2.97]) as compared to the febrile (VWF:CB 1.41 U/ml [1.27–1.93], p = 0.048) and the non-febrile control group (VWF:CB 1.15 U/ml [0.98–1.21], p<0.001). VWF:Ag tended to be higher in children with FS compared to both control groups. Multivariate regression analysis revealed FS and copeptin as major determinants of VWF:CB.

**Conclusions:**

Our results suggest that increased secretion of AVP in children with FS is associated with higher plasma levels of VWF parameters. Especially VWF:CB may serve as additional biomarker in the diagnosis of FS.

## Introduction

Febrile seizures (FS) are the most common type of seizures in children aged between 6 months and 5 years and occur in about 2–5% of children in the United States and Western Europe [1–4]. FS are defined as seizures occurring in childhood associated with fever that is not caused by an infection of the central nervous system [5]. Simple and complex febrile seizures are distinguished according to age at onset, frequency, duration and type of seizures. FS are normally considered benign with no impairment of cognitive outcome [6], but there is some evidence that children with a history of FS have a slightly higher risk to develop epilepsy [7, 8]. In some of these cases, genetically determined epilepsy manifests by propensity for febrile seizures.

Differentiation between febrile seizures and non-ictal events associated with fever such as shivering or dizziness is challenging. Therefore, precise diagnosis of FS after paroxysmal episodes associated with fever is often hindered by the lack of an objective biomarker. We investigated the diagnostic value of copeptin as a serum-biomarker for FS [9]. Copeptin is the C-terminal part of the arginine vasopressin (AVP) prohormone. Quantification of AVP can be difficult, whereas copeptin is stable in plasma and serum and thus has been recognized as a robust marker of AVP secretion [10–12]. In a previous study comprising a cohort of 161 children between 6 months and 5 years of age, presenting at the emergency department with the diagnosis of FS or an acute febrile infection without seizures, we measured serum copeptin levels in addition to standard clinical, neurophysiological, and laboratory assessments. We demonstrated that circulating copeptin was significantly higher in children with FS than in febrile controls. In a multivariable regression model, FS were the major determinant of serum copeptin independently of clinical and baseline laboratory indices [9].

In addition to the diagnostic value of copeptin in FS, we were wondering if AVP release in children with FS has any biological function. AVP is known to raise plasma levels of von Willebrand factor (VWF) parameters [13]. VWF is a multimeric plasma glycoprotein that maintains hemostasis by binding to platelets and collagen, and as carrier for factor VIII [14]. Upon AVP release, VWF is secreted from endothelial Weibel Palade bodies. This freshly secreted VWF is especially characterized by high molecular weight multimers. VWF analysis comprises measurement of VWF antigen (VWF:Ag) and VWF collagen binding activity (VWF:CB). The latter is used to detect these VWF high molecular weight multimers which are mainly responsible for collagen and platelet binding [15]. In addition, VWF multimeric analyses can be performed to investigate the triplet structure of VWF and the loss of high molecular multimers.

In this study, we hypothesized that VWF parameters, and here especially VWF:CB, are increased in children after FS compared to febrile or non-febrile controls due to an elevated AVP secretion.

## Methods

We conducted a prospective cross-sectional study at the Department of Pediatrics and Adolescent Medicine, University of Freiburg, Germany. The study was registered at “Deutsches Register Klinischer Studien”(No. DRKS00006251). The Ethics Committee of the Medical Center—University of Freiburg approved the study protocol (EK-Freiburg 137/14).

We investigated children aged between 6 months and 5 years who presented at our emergency department (ED) between 06/2014 and 03/2017. Inclusion criteria were defined as presentation with a febrile infection (temperature >38.0°C) or with a characteristic and unequivocal history of a recent FS (within 4 hours), and medical indication for a blood sampling at the ED due to the febrile infection or the FS. There was a medical indication for blood sampling in case of fever over several days with suspicion of a bacterial infection, a reduced general condition of the child, fever with no clear focus found, or the occurrence of a FS. FS were defined as a convulsive event associated with elevated body temperature (>38°C) without a previous history of afebrile seizures [5]. An experienced pediatrician reviewed the medical files of the enrolled infants and confirmed the diagnosis. Children with febrile infections were allocated to our primary control group (febrile control group). These children did not have a previous history of febrile or afebrile seizures. As a second control group, we included children who presented at our interdisciplinary outpatient clinic without fever or febrile seizures and without a previous history of seizures (non-febrile control group). These children had a medical indication for a blood sample due to a diagnostic intervention. Parental informed consent was obtained prior to blood sampling for the study. In children with FS, we performed the blood sampling in the emergency setting and obtained parental consent afterwards. Serum and citrated blood were centrifuged and serum and plasma were stored at -20° C and -80° C, respectively. Blood samples taken from infants with FS, whose parents declined study participation, were not analyzed.

For serum copeptin analysis, at least 0.5 ml of whole blood was required. Copeptin measurements were performed using the BRAHMS Kryptor Compact Immunoanalyzer (Thermo Scientific Brahms GmbH, Hennigsdorf, Germany). The lower detection limit of copeptin was 0.9 pmol/l, and the functional assay sensitivity (20% inter-assay coefficient of variance (CV)) was 2 pmol/l. The inter-assay precision was <7% CV at 5 pmol/l and <4% CV at 100 pmol/l.

As VWF parameters, we performed VWF:Ag-, VWF:CB- and VWF multimeric structure analyses. VWF:Ag, VWF:CB, and VWF multimeric structure analyses were investigated, as described previously [16]. Briefly, VWF:Ag was measured in sodium citrate plasma using an in house ELISA [17]. Collagen type I was immobilized on a microtiter plate. VWF:CB in plasma was measured photometrically via the ELISA technique. We calculated ratios of VWF:CB/VWF:Ag (normal ≥0.7), which reflect the biological capacity of the available VWF to bind to collagen. VWF multimers were separated on sodium dodecyl sulfate–agarose gel and blotted on a polyvinylidene fluoride membrane to assess the high molecular weight multimers. VWF was determined using appropriate primary and secondary antibodies and 3.30-diaminobenzidin/cobalt chloride. Standard human plasma was used as a control.

Clinical variables such as age, sex, body weight, body temperature, clinical characteristics of the febrile infection, duration and characteristics of seizures, and the amount of time between the episode and the sampling of blood were collected. Time of onset and duration of seizures were recorded according to the reports of parents. The laboratory testing in children with FS comprised analyses of hematocrit (HCT), white blood cell count (WBC) and serum sodium, determination of C-reactive protein (CRP) and blood gas analysis.

Descriptive data analyses were performed by calculation of absolute frequencies and percentages. Non-parametric data were analyzed as median with interquartile range (IQR) and compared using the Mann-Whitney U test. Statistical analyses were performed with SPSS 25.0 (IBM Corp., Armonk, NY). Univariate and multivariate regression analyses were applied to evaluate the effect of FS, fever, and various laboratory parameters (CRP, copeptin, HCT, WBC, and sodium) on levels of VWF:CB. A similar multivariable regression approach was used to explore the effect of various parameters (pH, base excess, sodium, CRP, HCT, WBC, body temperature at ED, duration of seizure, time between the episode and the sampling of blood) on VWF:CB in children with FS. Multivariate regression analysis was performed with a backward selection procedure with p = 0.1. Additionally, a Spearman’s correlation analysis between levels of copeptin and VWF:CB was performed. A p-value of <0.05 was considered statistically significant. Post-hoc power analysis was performed using the G*Power software [18].

## Results

Blood sampling was performed in 56 children: 30 children with FS, 10 children in the febrile control group, and 16 children in the non-febrile control group. All children had a medical indication for blood sampling and informed consent of parents was obtained. The diagnosis of FS was confirmed by an experienced pediatrician. Two children of our non-febrile control group were excluded due to an elevated CRP (CRP 25 mg/l) with the suspicion of an underlying infection in one child, and the diagnosis of a mild inherited von Willebrand disease in another child. In the remaining 54 children serum copeptin was measured. Citrated blood samples to analyze VWF parameters were collected in 15 children with FS, in 9 children with fever without febrile seizure and in 14 children without fever.

Children with FS did not differ in sex, age, body weight and temperature on admission from our febrile control group. Children of the non-febrile control group were older (median age of 4.3 years, IQR 2.2–4.8 years) compared to the FS group (median age of 1.7 years, IQR 1.5–2.5 years) and the febrile control group (median age of 1.4 years, IQR 1.3–2.7 years). The most common cause of fever in the FS group were infections of the upper airways in 17 children (56.7%) and fever of unknown origin in 8 children (26.7%). Four children (13.3%) showed complex febrile seizures. Clinical variables of all children are summarized in Table 1.

**Table 1:** Clinical variables of all children.

	Febrile seizures (n = 30)	Febrile control group (n = 10)	Non-febrile control group (n = 14)
**Age (years)**	1.7 [1.5–2.5]	1.4 [1.3–2.7]	4.3 [2.2–4.8]***
**Sex (♀/♂)**	14/16	4/6	7/7
**Body weight (kg)**	12 [11–12.4]	11.8 [10.6–14.4]	14 [12.7–18.5]***
**Body temperature (°C)**	38.8 [38.4–39.2]	39.1 [38.4–39.9]	36.9 [36.7–37.2]***
**Source of fever, n (%)**			
**Infection of upper airways**	17 (56.7%)	2 (20%)	NA
**Bronchitis**	1 (3.3%)	1 (10%)	NA
**Pneumonia**	0 (0%)	5 (50%)	NA
**Otitis media**	1 (3.3%)	0 (0%)	NA
**Gastroenteritis**	1 (3.3%)	0 (0%)	NA
**Stomatitis**	1 (3.3%)	0 (0%)	NA
**Exanthema subitum**	1 (3.3%)	0 (0%)	NA
**Lymphadenitis colli**	0 (0%)	1 (10%)	NA
**Pharyngitis**	0 (0%)	1 (10%)	NA
**No clear focus found**	8 (26.7%)	0 (0%)	NA
**Duration of seizure (min)**	2 [1–6]	NA	NA
**Time to blood sampling (min)**	85 [60–120]	NA	NA

Data are listed as median and IQR

*p <0.05,

**p <0.01, and

***p <0.001 for comparison between febrile seizures and controls

Compared to the febrile control group, laboratory testing revealed higher pH (FS: median pH 7.4, IQR 7.39–7,44; febrile control group: median pH 7.37, IQR 7.35–7.38; p = 0.085), lower base deficit (BE) (FS: median BE -4.4 mmol/l, IQR -5—-3.6 mmol/l; febrile control group: median BE-3 mmol/l, IQR -3.9—-2.3 mmol/l; p = 0.232) and significantly lower CO_2_ (FS: median CO_2_ 31.95 mmHg, IQR 28.1–35.33 mmHg; febrile control group: median CO_2_ 39.3 mmHg, IQR 37.3–44.2; p = 0.022) in children with FS. Febrile controls exhibited significantly higher CRP levels (CRP 11.5 mg/l, IQR 8.25–45.75 mg/l) compared to children with FS (CRP 5 mg/l, IQR 0–7 mg/l; p = 0.016) at presentation and were more frequently diagnosed with lower than upper respiratory tract infections (Table 2).

**Table 2:** Laboratory data of all children.

	Febrile seizures (n = 30)	Febrile control group (n = 10)	Non-febrile control group (n = 14)
**Copeptin (pmol/l)**	24.73 [13.65–68.65]	5.66 [4.15–8.07]**	4.78 [3.33–5.3]***
**VWF:Ag (U/ml)**	2.11 [1.29–2.51]	1.48 [1.34–1.68]	0.8 [0.69–1.0]***
**VWF:CB (U/ml)**	2.29 [1.88–2.97]	1.41 [1.27–1.93]*	1.15 [0.98–1.21]***
**Ratio VWF:CB/VWF:Ag**	1.32 [1.2–1.4]	0.88 [0.86–1.23]	1.38 [1.26–1.46]
**VWF multimers**	normal	normal	normal
**pH**	7.40 [7.39–7.44]	7.37 [7.35–7.38]	NA
CO_2_ (mmHg)	31.95 [28.1–35.33]	39.3 [37.3–44.2]*	NA
**BE (mmol/l)**	-4.44 [-5- -3.6]	-3 [-3.9- -2.3]	NA
**Sodium (mmol/l)**	136.5 [135–138]	135 [134–138]	139 [138.5–140]
**CRP (mg/l)**	5 [0–7]	11.5 [8.25–45.75]*	negative*
**WBC (G/l)**	11.94 [10–15.71]	10.85 [9.75–12.9]	8.9 [6.7–11.5]*
**HCT (%)**	34.3 [33–35.2]	33.5 [30.65–34.38]	36 [34–37]

Data are listed as median and IQR

VWF parameters were performed in 15 children with FS, in 9 children of the febrile control group and in 15 children of the non-febrile control group

*p <0.05,

**p <0.01

***p <0.001 for comparison between febrile seizures and controls

**Fig 1:**
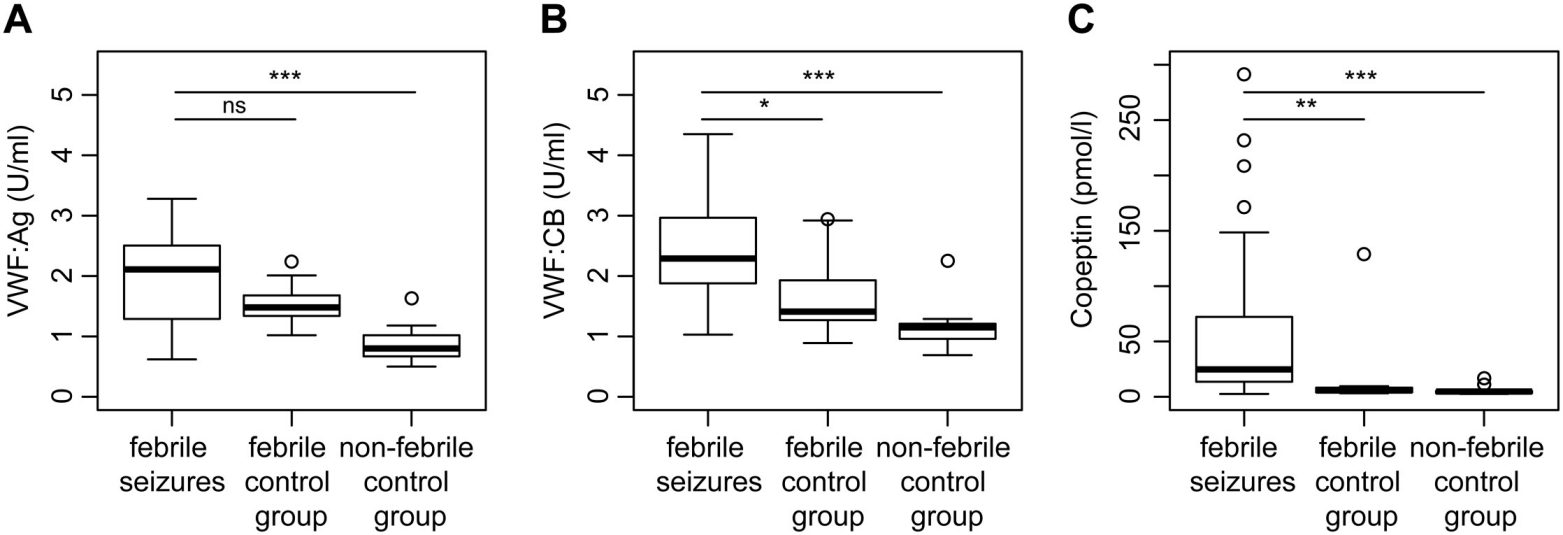
A) VWF:Ag, B) VWF:CB and C) serum copeptin levels in children with FS compared to febrile and non-febrile controls. Medians and interquartile ranges are also presented. Between-group comparisons were performed with Mann-Whitney U test. ns = not significant, *p <0.05, **p <0.01, and ***p <0.001 for comparison between febrile seizures and controls.

Serum copeptin levels were significantly higher in children with FS (median 24.73 pmol/l, IQR 13.65–68.65 pmol/l) compared to the febrile control group (median 5.66 pmol/l, IQR 4.15–8.07 pmol/l; p = 0.002) and the non-febrile control group (median 4.78 pmol/l, IQR 3.33–5.3 pmol/l; p<0.001). Regarding VWF parameters, we observed that VWF:CB was significantly higher in children with febrile seizures (median 2.29 U/ml, IQR 1.88–2.97 U/ml) compared to the control groups (febrile control group: median 1.41 U/ml, IQR 1.27–1.93 U/ml; p = 0.048; non-febrile control group: median 1.15 U/ml, IQR 0.98–1.21 U/ml; p<0.001). VWF:Ag was higher in children with FS (median 2.11 U/ml, IQR 1.29–2.51 U/ml) compared to the febrile control group (median 1.48 U/ml, IQR 1.34–1.68 U/ml; p = 0.263) and the non-febrile control group (median 0.8 U/ml, IQR 0.69–1.0 U/ml; p<0.001) (Fig 1).VWF multimeric structure analyses and the ratio of VWF:CB/VWF:Ag were normal in all children.

Univariate regression analysis revealed FS (*beta* 0.602, p<0.001) and copeptin (*beta* 0.486, p = 0.002) as major determinants of increased levels of VWF:CB (see Table 3). Backward selection of multivariate regression analysis showed FS and copeptin as only significant variables of increased levels of VWF:CB. Thus, multivariate regression analysis in children with febrile seizures did not reveal further significant determinants.

**Table 3:** Univariate regression analysis.

n = 54	R^2^	Beta	p-value
**Copeptin**	0.236	0.486	0.002
**FS**	0.363	0.602	0.000
**Fever**	0.005	-0,071	0.673
**Sodium**	0.079	-0.281	0.108
**CRP**	0.023	0.152	0.361
**HCT**	0.007	0.086	0.613
**WBC**	0.040	0.199	0.238

Univariate regression analysis was performed using VWF:CB as the dependent variable

**Fig 2:**
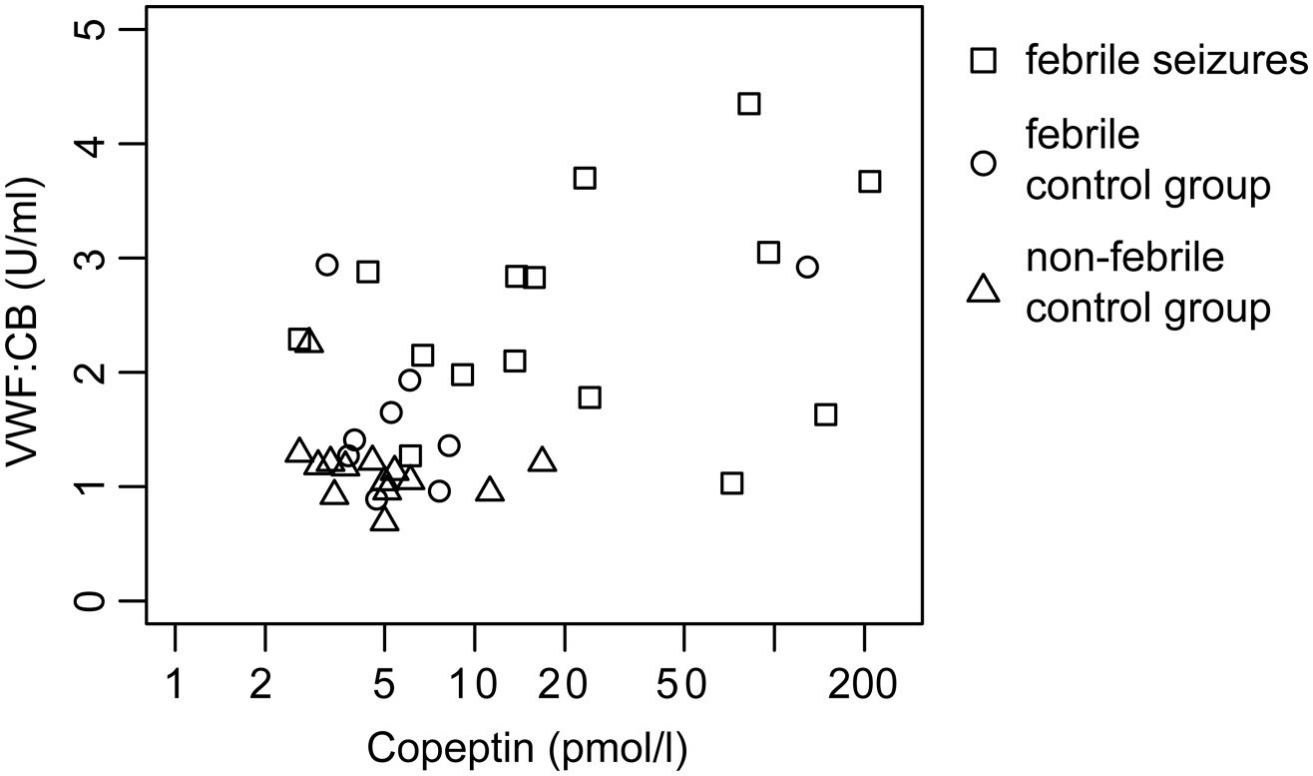
Correlation between copeptin and VWF:CB. X-axis is displayed logarithmically.

Correlation analysis of levels of copeptin and VWF:CB revealed a significant correlation (correlation coefficient 0.325, p = 0.046) (Fig 2).

For comparison of the FS group and the febrile control group, post-hoc power analysis revealed 43% power for evaluation of the observed differences in copeptin levels und 56% power for the differences in VWF:CB. Regarding the non-febrile control group, we reached 84% power for comparison of copeptin levels and 99% power for VWF:CB.

## Discussion

In this study, we investigated levels of serum copeptin and VWF parameters in children with FS compared to febrile and non-febrile controls. We detected significantly higher levels of serum copeptin and VWF:CB in children with FS compared to the control groups. Multivariate regression analysis revealed FS and copeptin as the only significant determinants of levels of VWF:CB among various different clinical and biochemical parameters.

The exact pathophysiological mechanism of FS remains unclear. There is some evidence that AVP plays an important role in thermoregulation and the development of FS [19, 20]. Further, preclinical data showed that hyperthermia causes respiratory alkalosis with consequent brain alkalosis and seizures [21], and clinical data showed an association of FS and systemic respiratory alkalosis [22]. In this context, respiratory alkalosis was identified as a major determinant of plasma copeptin levels in vivo [23]. In our previously performed clinical study, we could not confirm an association of FS and systemic respiratory alkalosis but identified copeptin as a potential biomarker for FS [9]. In the here presented study, serum copeptin levels in children with FS were significantly higher compared to children with febrile infections without seizures as well as compared to children without fever pointing out that copeptin may serve as specific biomarker for FS and does not increase due to stress in the context of febrile infections. However, we did not find an association to systemic respiratory alkalosis. Thus, the origin of copeptin release upon FS remains to be investigated.

AVP is known to raise plasma levels of VWF parameters [13]–mainly due to an increased secretion from endothelial Weibel Palade bodies. Measurement of VWF:CB is a very sensitive test to detect the freshly secreted VWF due to the content of high molecular weight multimers. Thus, as response to AVP, the increase in VWF:CB is higher than the increase in VWF:Ag reflecting this selective release of very high molecular weight multimers with greater functionality and thus greater capacity to bind to collagen [24]. Our VWF:CB assay uses Collagen type I, so that this assay is even more precise to measure VWF:activity and to detect VWF high molecular weight multimers than other VWF:activity tests [17]. This explains our results showing significantly higher VWF:CB in children with FS compared to both control groups as response to the AVP release. Thus, VWF:CB may serve as additional biomarker to adjust the diagnosis of FS.

In our cohort, levels of VWF:Ag were increased in children with FS and in children of the febrile control group compared to the non-febrile controls, reflecting that VWF is an acute-phase reactant so that secretion rates increase in stressful status such as acute infection [14]. Levels of VWF:Ag can also be influenced by other factors such as age, race, stress or blood group [25]. Therefore, assessments measuring WVF:Ag are limited as they only address the total level of WVF protein [26, 27]. Children of our febrile control group showed more severe infections as indicated by significantly higher CRP values and higher percentage of pneumonia compared to upper respiratory tract infections in the FS group (see Tables 1 and 2). We only included children in our study with an indication for blood sample at the ED due to the febrile infections and blood samples are usually not taken in children with uneventful febrile infections. This may explain the elevated levels of VWF:Ag in both the FS group and the febrile control group compared to the non-febrile controls.

## Conclusions

Our results show for the first time increased VWF parameters, especially VWF:CB, in children with FS indicating increased activated primary hemostasis. Increased VWF parameters are associated with an increased secretion of copeptin, a surrogate marker of AVP. Thus, copeptin and VWF parameters, especially VWF:CB, may serve as additional biomarkers in the diagnosis of FS. Future studies may investigate the underlying mechanism of AVP/copeptin release in FS as well as the causal link between increased AVP/copeptin release and increased WVF parameters.
